# United Kingdom: transfers of genomic data to third countries

**DOI:** 10.1007/s00439-018-1921-0

**Published:** 2018-08-03

**Authors:** M. J. Taylor, S. E. Wallace, M. Prictor

**Affiliations:** 1 HeLEX@Melbourne, Melbourne Law School, University of Melbourne, Carlton, Australia; 2 Population and Public Health Sciences, Department of Health Sciences, University of Leicester, Leicester, UK; 3 Nuffield Department of Population Health, Centre for Health, Law and Emerging Technologies (“HeLEX”), University of Oxford, Oxford, UK

## Abstract

In the United Kingdom (UK), transfer of genomic data to third countries is regulated by data protection legislation. This is a composite of domestic and European Union (EU) law, with EU law to be adopted as domestic law when Brexit takes place. In this paper we consider the content of data protection legislation and the likely impact of Brexit on transfers of genomic data from the UK to other countries. We examine the advice by regulators not to rely upon consent as a lawful basis for processing under data protection law, at least not when personal data are used for research purposes, and consider some of the other ways in which the research context can qualify an individual’s ability to exercise control over processing operations. We explain how the process of pseudonymization is to be understood in the context of transfer of genomic data to third parties, as well as how adequacy of data protection in a third country is to be determined in general terms. We conclude with reflections on the future direction of UK data protection law post Brexit with the reclassification of the UK itself as a third country.

## Introduction

The Data Protection Act 2018 (DPA 2018) is the United Kingdom’s (UK’s) third generation of data protection legislation. Its main provisions came into force on 25 May 2018; the same day as the enforcement date for the General Data Protection Regulation (GDPR).

While the UK remains a member of the European Union (EU), the GDPR will apply directly. When the UK leaves the EU, the GDPR will be incorporated within domestic law. This will happen with the operation of the European Union (Withdrawal) Act 2018, so the DPA 2018 did not need to replicate the GDPR itself. However, the DPA 2018 is the vehicle through which the UK has specified how various aspects of the GDPR will apply in the UK (DPA 2018, s. 22), and takes advantage of the opportunities provided by the GDPR for domestic derogation. When the UK leaves the EU, the concept and membership of “third countries” will change. We consider this later when examining the issues of “Adequacy” and “Future Directions”.

The DPA 2018 and the GDPR—collectively referred to, with relevant regulations, as “the data protection legislation” [DPA 2018, s. 3(9)]—protect individuals in relation to the processing of personal data in the UK (GDPR, art. 3). Together, they will establish the legal data protection framework applicable to third country transfers (i.e., countries outside of the EU and European Economic Area (EEA) Member States) from the UK, even after the UK’s withdrawal from the EU.^1^ The material scope (subject matter) and territorial scope (where it applies) are described in the Regulation. In this article, we consider how the new data protection legislation impacts upon UK transfers to “third countries”, with particular attention to transfers for reasons of scientific research.

The material scope of data protection legislation extends to the processing of personal data by automated (e.g., digital) means, where personal data forms part of a filing system,^2^ and to some manual unstructured processing.^3^ There are exceptions—e.g., data processed in the course of a purely personal or household activity [GDPR, art. 2(2)(c); DPA 2018, s. 21(3)]—but otherwise the legislation is intended to establish a far-reaching scheme of data protection, covering the broadest range of personal data, and processing activity. Caught within the material scope are data concerning health (“health data”),^4^ and genetic data processed for any purpose including transfer to third countries for health, research,^5^ or commercial purposes. Genetic data means:

“[P]ersonal data relating to the inherited or acquired genetic characteristics of a natural person which give unique information about the physiology or the health of that natural person and which result, in particular, from an analysis of a biological sample from the natural person in question” [GDPR, s. 4(13)].

This definition of genetic data is broad enough to include genomic data—we will use the former term throughout. The transfer of genetic data may be accompanied by the transfer of other (e.g., biographic or phenotypic) data. When relating to an identifiable person, such data will also typically fall within the material scope of the data protection legislation. While not the focus of our attention, many of our comments will also be applicable to these data.

Any processing of personal data must satisfy minimum requirements. Where processing is understood to pose particular risks, there will be a proportionate increase in the expectations of controllers to implement appropriate and effective measures, and to demonstrate compliance (see esp. Recital 74–78). The processing of personal data concerning health, and genetic data, represent examples where special attention should be paid to the likelihood and severity of the risks to the rights and freedoms of natural persons (Recital 75). Where processing involves these and other special categories of data, there is a presumption that such processing is prohibited unless one of several specific conditions applies. In the UK, the Health Research Authority (HRA) has issued guidance on the conditions that university researchers should ordinarily rely upon as a lawful basis for processing under data protection law. This legal basis should not ordinarily be consent (Health Research Authority 2018a).

The territorial scope of the GDPR extends to processing of the personal data of data subjects in the EU, by a controller or a processor not established in the Union, where related to the offer of goods or services, or monitoring of behaviour within the Union [GDPR, art. 3(2)]. To the extent that such processing involves any transfer of genetic data (e.g., through the targeting of genetic testing services to data subjects in the EU), it falls within the scope of the GDPR.

The DPA 2018 contains a number of provisions relevant to international genomic data sharing. In the UK, the text of DPA 2018 is supplemented by explanatory notes issued by Parliament. The HRA has a responsibility to issue guidance on how legal requirements apply to research in England;^6^ the Information Commissioner’s Office (ICO) is the relevant data protection supervisory authority in the UK; and other organizations, such as the Medical Research Council, issue guidance that is directed not only to researchers whom they fund, but also has broad value to the research community.

Thus, a comprehensive overview of the UK position can be constructed from the data protection legislation and the accompanying explanatory notes, as well as from issued guidance. Owing to its novelty, there is relatively little academic critique of the data protection legislation in the UK. It should be noted, however, that this legislation does not exhaust all law relevant to genetic data sharing. While we focus on data protection legislation, we supplement our analysis with reference to other law; e.g., legal duties of confidentiality, where appropriate.

## Consent

The bottom line is that, under UK data protection legislation, consent is not a prerequisite for the lawful transfer of genetic data to a third country; at least, not if the transfer is necessary for research or clinical purposes. Transfer for commercial purposes (including, for example, direct-to-consumer genetic test data), which is unnecessary for either research or clinical purposes, may require “explicit consent” (GDPR, art. 9).

Lawful processing of personal data requires that one of the conditions set out in Article 6(1) of the GDPR is met. The first available condition is that the data subject has given consent to the processing of personal data for one or more specific purposes. When the Regulation was being debated, the research community (including in the UK) expressed concern that the stringent definition of consent under the GDPR [GDPR, art. 4(11)] might make it difficult to obtain valid consent. Particularly problematic was the case where future uses of personal data were not known at the time of collection with any specificity. Existing practice included asking participants to provide consent in broad terms.^7^

The response was, through Recital 33, to recognize that “data subjects should be allowed to give their consent to certain areas of scientific research when in keeping with recognised ethical standards for scientific research”. In practice, questions regarding the sufficiency of that response, particularly in relation to an “explicit” consent,^8^ have largely been rendered moot for now in the UK by guidance from the ICO^9^ and the HRA. The HRA website makes clear that:

“For the purposes of the GDPR, the legal basis for processing data for health and social care research should NOT be consent. This means that requirements in the GDPR relating to consent do NOT apply to health and care research” (Health Research Authority 2018a).

HRA guidance is that data subjects are told by any public sector controller, including a university [DPA 2018, s. 7(1)(a); Freedom of Information Act 2000 (FOI Act), sch. 1, pt IV, para. 53(1)(b)] or National Health Service (NHS) organization, that processing of personal data for research purposes is in the public interest (Health Research Authority 2018b): to be demonstrated by following the UK Policy Framework for Health and Social Care Research (Health Research Authority 2018c). This, rather than consent, is advised to be the most appropriate way for a public sector organization to demonstrate a lawful basis to process data (a private sector organization may rely upon the “legitimate interests” condition in Article 6).

Additional requirements must be met under data protection legislation in relation to special categories of data, such as genetic data. Here, if the transfer is necessary for research purposes the advice is to rely upon the fact that processing for approved (DPA 2018, s. 19) purposes is expressly permitted (Health Research Authority 2018b), rather than upon “explicit consent”.

The net result is that consent is not understood to be a legal requirement for the processing of personal data, including the international transfer of genetic data to a third country, so far as that transfer constitutes an aspect of processing necessary for research. At least, it is not a general requirement of data protection legislation. Although, special data protection rules apply to third country transfers, which means that—in some cases—consent might still be necessary where there is no alternative. This is discussed under the heading “Adequacy” below. The point to note is that, even here, consent is not the only way for a controller to demonstrate compliance with data protection law; nor even here, would it be preferred.

Other legal requirements, beyond data protection legislation, apply to those in the UK wanting to transfer genetic data to a third country. For example, where research activity involves processing identifiable genetic data, collected originally from patients in England and Wales as part of the delivery of care, then any disclosure of those data outside the direct care team may constitute a breach a duty of confidentiality [General Medical Council 2017, (77–80)]. In such circumstances, an application may be made for the duty to be set aside if it is impracticable to either seek consent for the disclosure, or for the data to be anonymized before disclosure [Health Service (Control of Patient Information) Regulations 2002; National Health Service Act 2006, s. 251; see also Health Research Authority 2018d].

Even if the processing of identifiable genetic data takes place without an individual’s (explicit) consent, it does not follow that there are no attendant privacy protections. Data protection legislation alone establishes a number of privacy protections in the UK that apply irrespective of whether an individual has given consent to the transfer.

## Privacy

Privacy and data protection are linked, but distinct concepts. If privacy is “established by norms regulating access to individuals or groups of individuals” (Taylor 2012, 25; see also Laurie 2002, 6), then data protection legislation works in the UK to uphold norms of data separation and exclusivity by recognizing, and limiting, data subject rights to control access to information in various ways. There are norms applicable to any processing, and there are particular norms established in relation to processing of special kinds. For example, any processing should uphold the right to respect for private and family life, and the right to protection of personal data, as set out in Article 7 and Article 8 of the EU Charter respectively [Charter of Fundamental Rights of the European Union (2000/C 364/01)]. If genetic data are being processed for scientific and historical research purposes (or archiving in the public interest) (European Commission 2018), then processing will also be subject to the specific requirement in Article 89(1) GDPR for appropriate safeguards to protect fundamental rights and freedoms.

Some of the most celebrated changes to data subject rights introduced, or at least strengthened, by the GDPR relate to data portability and to erasure (“the right to be forgotten”). Each may be perceived as relevant to the transfer of genetic data to a third country, and thus requires brief coverage.

### The right to data portability

Data protection legislation gives data subjects the right to data portability:

“The data subject shall have the right to receive the personal data concerning him or her, which he or she has provided to a controller, in a structured, commonly used and machine-readable format and have the right to transmit those data to another controller without hindrance from the controller to which the personal data have been provided” [GDPR, art. 20(1)].

This right will be limited in the context of genetic data in two important respects. First, it only extends to data provided by the data subject. This does not “include data that are created by the data controller (using the data observed or directly provided as input)” (Article 29 Data Protection Working Party 2017, 10). Where a data subject has provided biological material, and a controller has derived genetic data through analysis of that material, it seems very unlikely that the right to data portability will extend to those derived data. Second, the right only applies where processing is on the basis of consent or contract [GDPR, art. 20(1)(a)]. Where a data controller relies upon a lawful basis for the processing other than consent, as recommended by the HRA as discussed above, then the data subject’s right to data portability does not apply.

### The right to erasure

Data protection legislation provides data subjects with the right to request erasure of personal data (GDPR, art. 17; DPA 2018, s. 47). This extends to genetic data held by a controller where related to a data subject in identifiable form. HRA guidance to researchers in the UK notes that this right does not apply when data are processed for research purposes if erasure would “render impossible or seriously impair the achievement of the objectives of that processing”, and if appropriate safeguards are in place [GDPR, art. 17(3)(d)]. Examples provided by the HRA include where:

“For research integrity and participant accountability reasons, the research may not meet the standards expected if data were to be erased, and therefore the research activity could be seriously impaired.

Erasing data when a database has been locked for analysis would seriously impair achievement of the purposes of a research activity (as would rectification when the research is based on a snapshot of time)” (Health Research Authority 2018e).

This would seem to cast the net wide and to indicate that the right to erasure is significantly qualified in the research context. It is emphasized that this qualification only applies where appropriate safeguards are in place.^10^ In particular, in the UK, data protection legislation specifies that processing does not satisfy the requirement in Article 89(1) for appropriate safeguards if the processing “is likely to cause substantial damage or substantial distress to a data subject” [DPA 2018, s. 19(2)]. One cannot, therefore, rely upon any research exemption from data erasure where doing so would likely cause substantial damage or distress.

If a controller does erase data, then the explanatory notes to the DPA 2018 indicate that there is a “duty on the controller to inform the competent authority from where the data originated (if different) and to alert any recipients of the data. This is particularly important if data have been transferred internationally” (Explanatory Notes to DPA 2018, para. 203). This is presumably only intended to apply where data are erased due to inaccuracy or other infringement of data protection legislation (see GDPR, Recital 47, art. 16; DPA 2018, s. 47).

## Security measures

Data protection legislation in the UK requires that personal data are processed in a manner

“that ensures appropriate security of the personal data, including protection against unauthorised or unlawful processing and against accidental loss, destruction or damage, using appropriate technical or organisational measures (‘integrity and confidentiality’)” [GDPR, art. 5(1)(f); see also GDPR, Recitals 39 and 83, art. 32].

A controller will be expected to ensure an appropriate level of security; appropriate to the risk, “taking into account the state of the art, the costs of implementation and the nature, scope, context and purposes of processing” [GDPR, art. 32(1)]. Data availability is recognized as an important part of data security, alongside data integrity and confidentiality [GDPR, art. 32(1)(b)]. In practice, no single measure is likely to satisfy the requirement for appropriate protection and meet the principle of privacy by design. User authentication and the technical means to enable the exercise of data subject rights will be as important as the organizational and technical means of protecting data from accidental loss, destruction, or damage.

Pseudonymization and encryption are recognized by data protection law as potentially appropriate privacy protective measures. It is worth pausing briefly to explain what is meant by pseudonymization in this context, and to consider its significance for genetic data:

“‘[P]seudonymization’ means the processing of personal data in such a manner that the personal data can no longer be attributed to a specific data subject without the use of additional information, provided that such additional information is kept separately and is subject to technical and organisational measures to ensure that the personal data are not attributed to an identified or identifiable natural person” [GDPR, art. 4(5)].

This definition has to be placed within the context of the material scope of the GDPR. Pseudonymization is a process applied to personal data. It is intended to reduce the risks to data subjects and to help controllers and processors to meet responsibilities (GDPR, Recital 28). Data that have been through a process of pseudonymization will only continue to fall within the scope of the GDPR if they remain personal data. This depends upon data continuing to relate to an identifiable person. Recital 26 makes it clear that when determining whether a natural person is identifiable

“account should be taken of all the means reasonably likely to be used, such as singling out, either by the controller or by another person to identify the natural person directly or indirectly. To ascertain whether means are reasonably likely to be used to identify the natural person, account should be taken of all objective factors, such as the costs of and the amount of time required for identification, taking into consideration the available technology at the time of the processing and technological developments” (GDPR, Recital 26).

This means that, if all means reasonably likely to be used are taken into account, and the individual to whom the data relate is not identifiable, then the data will fall outside of the scope of the GDPR (Mourby et al. 2018). This is true even of genetic data, where there is a theoretical possibility of (re)identification, if the likelihood of identification is, in practice, sufficiently remote.

Pseudonymization is a process that can be implemented within the context of a data controller (GDPR, Recital 29). In this case, the controller will retain the means—which might reasonably likely be used—to relate the pseudonymized data to an identifiable person. If, however, the controller discloses “pseudonymized data” to a third party, and the data subject is no longer identifiable in the disclosed data from the perspective of the receiver, then those data will no longer be personal data—and will no longer fall within the scope of data protection legislation (Pormeister 2017).

For this reason, and in this context, it will be important to reserve use of the term pseudonymization to a process that results in data remaining personal after the process has been applied. If data are perturbed, redacted, or subject to other processing, and the outcome is that there are no means reasonably likely to be used to relate the data to an identifiable individual, then the data are more properly described as anonymized. This does not overlook the rich literature relating to the potential for re-identification of genetic data (e.g., Phillips and Knoppers 2016; Cai et al. 2015). That literature simply informs an understanding of what contextual controls would be necessary to establish that any risk of (re)identification, in the circumstances, has been sufficiently mitigated.

## Adequacy

Transfers of personal data outside the EU and EEA are subject to special controls (GDPR, art. 44) to ensure that personal data are only transferred into environments where they will continue to be subject to adequate protection; and the adequacy of data protection law in third countries is not to be assumed by controllers. For this reason, transfer is permitted only if: (a) the European Commission has decided that the third country ensures an adequate level of protection (Art. 45) (GDPR, Recitals 103–107 and 169)^11^; (b) the controller or processor has provided adequate safeguards (Art. 46) (GDPR, Recitals 108–110 and 114);^12^ or (c) one of the derogations for a specific situation is applicable.

Where a transfer cannot be based on (a) or (b), then Article 49(1) sets out eight situations in which (c) derogation is possible. The first is where

“the data subject has explicitly consented to the proposed transfer, after having been informed of the possible risks of such transfers for the data subject due to the absence of an adequacy decision and appropriate safeguards” (GDPR, art. 49(1)(a)).

This derogation is not available to public authorities “in the exercise of their public powers” [GDPR, art. 49(3)]. This raises a question as to whether UK universities can rely upon explicit data subject consent to legitimate transfers to a third country (in cases where the provisions of Article 45 and Article 46 are inapplicable). As noted above, due to their status as public authorities [DPA 2018, s. 7(1)(a); FOI Act, sch. 1, pt IV, para. 53(1)(b)], UK universities are advised to establish, as a lawful basis for processing, that it is

“necessary for the performance of a task carried out in the public interest or in the exercise of official authority vested in the controller” [GDPR, art. 6(1)(e)].

Thus, if transfer is understood to be “in [the] exercise of their public powers”, then this might seem to render derogation unavailable due to a data subject’s explicit consent. In fact, we suggest that where the transfer is necessary for the performance of a task carried out in the public interest, but requires no specific power to be exercised, then it remains open to a public authority (otherwise unable to rely upon Article 45 or Article 46) to rely upon a data subject’s explicit consent to the transfer to a third country, where appropriate protection cannot be assured.

While we see this as a reasonable interpretation of the domestic legislation, even if public authorities could rely upon explicit consent, it does not follow that they should. It would seem preferable for a public authority—with the resources of a public authority—to provide adequate safeguards (consistent with Article 46), rather than to rely upon explicit consent to justify a derogation. Where it is genuinely impossible to do so, but there is nonetheless an important “public interest” reason for the transfer, then consent may still not be the most appropriate derogation to rely upon.

There is a specific derogation available where transfer is necessary “for important reasons of public interest” [GDPR, art. 49(1)(d)].^13^ If there really are important public interest reasons for the transfer, those could be specified in domestic law [DPA 2018, s. 18(1)]. If there is no important public interest reason for the transfer, then it may be inappropriate to ask people to agree to the flow of personal data into environments where adequate protection cannot be assured.

The DPA 2018 allows for secondary legislation to specify (for the purposes of GDPR, art. 49(1)(d)) circumstances in which the transfer of personal data to a third country or international organization is necessary for important reasons of public interest [DPA 2018, s. 18(1)].^14^ Unsurprisingly, given the recent nature of the legislation, no regulations have yet been passed relevant to the transfer of genetic data to third countries. This remains an option if existing alternative legal routes to transfer are insufficient to meet an important public interest need. In summary, adequacy could, and we suggest ordinarily should, be met without the need to resort to derogation on the basis of explicit consent.

Finally, we should briefly mention a point that we pick up again under “Future Directions”. As noted at the beginning of this section, (1) “adequacy” of protection need only be specifically addressed in relation to transfers out of the EU, and (2) data protection legislation in the UK incorporates the content of the GDPR. As a consequence, at the point of exit from the EU, controllers in the UK must continue to meet adequacy requirements in relation to transfers to countries outside of the Union. English law will continue to regard such international transfers as requiring special protection. All controllers in the Union will, of course, from that point regard the UK as a “third country”.

## Oversight

Oversight in the UK is expected to continue as in the past, but with some provisions strengthened as required by the GDPR and DPA 2018. Certainly, in response to the new legislation, and reflecting the increasing difficulties of informing an individual about all the ways personal data are being used, more emphasis is being placed on accountability. No longer can data protection issues be left to others. Organizations and their staff must take responsibility for knowing, for example, what data are being collected, who has access to the data, how they are being used, how long they can be kept, and under which consent data were obtained (Information Commissioner's Office 2018c).

While previously data controllers had to report their processing activities to a local data protection authority, the GDPR brings record keeping “in-house”. Public authorities and other organizations processing certain categories of data must appoint a dedicated Data Protection Officer (DPO) (GDPR, art. 37; Information Commissioner's Office 2018b). The DPO is the contact for those seeking assistance and needs to be knowledgeable about data protection issues, how data are being processed at their organization, and what oversight measures are in place or required to protect those data. Recital 97 of the GDPR stresses that DPOs, “whether or not they are an employee of the controller, should be in a position to perform their duties and tasks in an independent manner” to ensure unbiased oversight. If processing might pose a high risk to individuals, due to the nature of the data being gathered or technologies used, then the DPA 2018 requires the DPO to carry out a Data Protection Impact Assessment (DPIA) detailing the possible risks and how these will be managed (Information Commissioner's Office 2018a). Security measures must be continuously monitored and reviewed, and DPOs need to work with research governance officers in the NHS and universities to update existing research governance strategies. As in the GDPR, the DPA 2018 requires that any data breach must be reported “without undue delay, and … where feasible, not later than 72 h after becoming aware of it” [DPA 2018, s. 67(1)].

All details on measures taken, such as the contact details of DPAs, any DPIAs, and information on data breaches must be reported to the ICO. A certification scheme is being discussed by the new European Data Protection Board (EDPB) (replacing the Article 29 Group) to prove compliance to the GDPR; details have yet to be published. While currently a member, EU representatives stated recently that the UK will no longer be able to take part once it leaves the EU (Afifi-Sabet 2018; see also Murray 2017, 150). Whether this will continue to hold true over the coming months is unclear, but it no doubt impels the UK to focus on its own existing data protection mechanisms to ensure European compliance while still allowing flexibility for the future.

To this end, the DPA 2018 seeks to strengthen the existing, apparently effective, safeguards. The HRA has stated that it will not be adding any additional oversight mechanisms for processing health and social care data (Health Research Authority 2018g). Research Ethics Committee (REC) approval will still be required where applicable, and the Confidentiality Advisory Group (CAG) will continue to advise on operation of the legal gateway in England and Wales for disclosure of confidential patient information without consent [Health Service (Control of Patient Information) Regulations 2002; National Health Service Act 2006, s. 251; see also Health Service Authority 2018d].^15^ The ICO continues to publish guidance to assist organizations. However, there is concern that the UK could become more insular and less outward focused, thus limiting opportunities to look at new technological opportunities that will promote data sharing across borders for the public good; an initiative recommended recently by the OECD (Health Research Authority 2018g).

## Future directions

One of the most significant considerations regarding future governance of genetic data transfer to a third country is the impending exit from the EU. While not all of the likely consequences can be anticipated, some of the most obvious include: the UK becoming a “third country” for the rest of the EU and EEA; the consequent impact upon data flows to the UK from the rest of Europe; and the impact of disengaging UK supervisory authority from the EDPB. The UK has assured business that it recognizes the need to implement the GDPR to ensure work can continue as before, after Brexit. It has also indicated a wish to retain membership of the EDPB, so as to contribute to future policy decisions (Boffey 2018). However, this has not met with approval from EU Brexit negotiators. Michel Barnier has publicly stated that the UK will not be able to remain at the table after Brexit, as standards will be decided by EU member states, not “third parties” (Barnier 2018). Now the UK will need to “think” as a third country, in order to determine how transfers to it from the EU will comply with adequacy requirements. The UK could try to ensure that its laws meet EU requirements and maximize the chance of an adequacy finding by the Commission. This will take time. Meanwhile, the UK might seek to make it as easy as possible for controllers in the rest of Europe to account for appropriate safeguards (GDPR, art. 46). A contrary possibility is that the UK could decide to base its data protection standards on other jurisdictions. This might reflect, as in other areas of future UK law, a drift away from EU law. Some may consider it more important that agreements are set up with countries such as the United States, India, or China. Opinions vary widely and the EU, in its handling of the UK’s move to third party status, may contribute to a shift. Whether this will be detrimental is, of course, dependent upon one’s position on the best path for the UK to take (see e.g., de Hert and Papakonstantinou 2017). Because such decisions are in the future, care must be taken to ensure that there are no gaps which might allow the misuse of data sharing, as well as missed opportunities to participate in research studies that have the possibility of improving the lives of individuals.

The impact of the UK’s reclassification to “third country” status includes uncertainty regarding the protection available to UK citizens due to the territorial scope of the GDPR. As noted earlier, the GDPR extends to the processing of personal data by a controller or a processor not established in the Union, where related to the offer of goods or services, or the monitoring of behaviour within the Union. To the extent that such processing involves any transfer of genetic data, it is caught within the scope of the GDPR. While the UK is an EU member, organizations offering goods or services to British citizens will, when processing personal data, need to comply with the GDPR. Post Brexit, it is hard to see how the UK will be able to leverage compliance with such a requirement. UK organisations handling EU data will, however, still have to comply. In practice, this may reduce the protection available to UK citizens but not affect the regulatory burden on UK organisations offering genetic testing services into the Union.

To conclude, we reflect upon the fact that Brexit is expected to have no immediate impact on UK membership of the Council of Europe (CoE). There are responsibilities owed under the Convention for the Protection of Individuals with regard to Automatic Processing of Personal Data (ETS No.108) that will be unaffected by Brexit and reflect broadly similar principles to those contained in the GDPR. This should help mitigate the risks of drift. Also, continued membership of the CoE should mean the continued incorporation of the European Convention of Human Rights (ECHR) into domestic law through the Human Rights Act 1998. However, there have been calls in the past to repeal or at least to amend the Human Rights Act, and to withdraw from the ECHR. As with much of Brexit, currently there is no certainty, but it is generally recognized that the Human Rights Act has brought much needed protections. With the expected changes, the challenges that such changes will bring, and the possibility that the detail of data protection will drift from that of the UK’s European neighbours, an overarching human rights framework that is uniform across the UK and Europe will provide much-needed reassurance and possible avenues for redress. We must hope that such a framework, alongside individual project commitment to initiatives such as the Global Alliance for Genomics and Health (Global Alliance for Genomics and Health 2017), will help to maintain a level of consistency and uniformity, and avoid fragmentation of the governance essential to flows of genetic data in the public interest.

## Footnotes

1 Transfers to countries in the European Union are regulated in the same way as transfers of data within the UK. From a UK perspective, this will not change post-Brexit: the idea of a “third country” adopted by UK legislation is provided by the GDPR.

2 “Filing system” means any structured set of personal data which is accessible according to specific criteria, whether centralized, decentralized, or dispersed on a functional or geographical basis: GDPR, art. 4(6).

3 The scope of data protection legislation is extended in the case of public authorities in the UK, to include the manual unstructured processing of personal data: DPA 2018, s. 21(2).

4 “Data concerning health” means personal data related to the physical or mental health of a natural person, including the provision of health care services, which reveal information about his or her health status.

5 Recital 159 provides guidance on the scope of the research processing to which the Regulation is intended to apply. It is broad.

6 Under Care Act 2014, s. 111(6), the HRA must publish guidance on—(a) principles of good practice in the management and conduct of health and social care research; (b) requirements, whether imposed by enactments or otherwise, to which persons conducting health or social care research are subject.

7 E.g., the consent form used by UK Biobank invited people to indicate “permission for access to my medical and other health-related records, and for long-term storage and use of this and other information about me, for health-related research purposes (even after my incapacity or death)”: see Biobank (2018) Resources. See also Hallinan and Friedewald (2015).

8 GDPR, art. 9, raises the presumption that the processing of special categories of personal data, including genetic data, will be prohibited. The presumption is only rebutted if one of a number of specific conditions applies. While the first listed alternative under each of these articles is consent, Article 9 consent must be “explicit”.

9 “You are likely to need to consider consent when no other lawful basis obviously applies.” https://ico.org.uk/for-organisations/guide-to-the-general-data-protection-regulation-gdpr/consent/when-is-consent-appropriate/ Accessed 25 July 2018. “Even if individuals have consented to participate in the research, you may well find that a different lawful basis (and a different special category data condition) is more appropriate in the circumstances.” https://ico.org.uk/for-organisations/guide-to-the-general-data-protection-regulation-gdpr/consent/what-is-valid-consent/ Accessed 25 July 2018. This is consistent with the guidance given by the Article 29 Working Party (2018), p28–—.

10 For UK discussion of appropriate safeguards, see Health Research Authority (2018f).

11 On the challenges pertaining to such decisions, see Morrison et al (2017).

12 Where a group of undertakings, or a group of enterprises engaged in a joint activity, undertake the processing of personal data, then transfers between members may be governed by Binding Corporate Rules (BCR). BCR are approved by supervisory authorities (consistent with mechanisms set out in Article 63) provided that they meet a set of criteria specified in the GDPR. See GDPR, art. 47; see also Bowman and Gufflet (2017).

13 Article 49(4) enables Members States to create domestic law to specify important reasons of public interest, for the purposes of Article 49(1)(d). DPA 2018, s. 18(1), provides the Secretary of State with order making powers for this purpose.

14 This same section empowers a minister to specify when any transfer, not required by enactment, would not be necessary for important reasons of public interest.

15 In Scotland, one would approach the Public Benefit and Privacy Panel (PBPP) or local Caldicott Guardian; and in Northern Ireland, the Privacy Advisory Committee (PAC): Medical Research Council (2018).
